# Cryogenic Cooling in Wireless Communications

**DOI:** 10.3390/e21090832

**Published:** 2019-08-25

**Authors:** Tomasz G. Markiewicz, Krzysztof W. Wesołowski

**Affiliations:** Faculty of Electronics and Telecommunications, Poznań University of Technology, Polanka 3, 60-965 Poznań, Poland

**Keywords:** wireless communications, channel capacity, Shannon limit, cryogenics, thermal noise

## Abstract

Improving the capacity and performance of communication systems is typically achieved by either using more bandwidth or enhancing the effective signal-to-noise ratio (SNR). Both approaches have led to the invention of various transmission techniques, such as forward error correction (FEC), multiple-input multiple-output (MIMO), non-orthogonal multiple access (NOMA), and many, many others. This paper, however, focuses on the idea that should be immediately apparent when looking at Shannon’s channel capacity formula, but that somehow remained less explored for decades, despite its (unfortunately only in theory) limitless potential. We investigate the idea of improving the performance of communication systems by means of cryogenic cooling of their RF front-ends; the technique, although widely-known and used in radio astronomy for weak signal detection, has attracted limited interest when applied to wireless communications. The obtained results, though mainly theoretical, are promising and lead to a substantial channel capacity increase, implying an increase in spectral efficiency, potential range extension, or decreasing the power emitted by mobile stations. We see its applications in base stations (BSs) of machine-type communication (MTC) and Internet of Things (IoT) systems.

## 1. Introduction

Shannon’s formula for channel capacity, i.e.,

(1) $$C=B\log\left(1+\frac{S}{N}\right),$$

suggests two main methods that allow an increase of system capacity. The first is by enlarging the received signal power, *S*, which produces logarithmic gains in terms of *S*. The second allows linear gains with respect to changes of its value and corresponds to using larger amounts of spectral resources (here: *B* is bandwidth). Both the *signal* and *bandwidth* methods are intuitive. However, while transmitted (and as a result, received) power is subject to various regulations at governmental levels and can be increased only according to them, bandwidth in wireless communications is a resource that is indisputably scarce, which is even worse.

Looking again at Equation (1) immediately suggests that there is a third method of increasing the system capacity, which in our opinion has not attracted due attention. Explicitly listing the previous signal and bandwidth methods leaves us with the only possible letter on the right-hand side of Equation (1) that remains unused, i.e., *N*, and as a result, we refer to it as a *noise* method. The possibility of lowering the noise power itself in order to improve the capacity is not prevalent in the communications community, regardless of the considerable gains that might result. This is despite the fact that most of the attention is concentrated on the design of an RF receiver block featuring low noise figure (NF) values. In this paper, we investigate the impact of limiting the overall noise in order to improve the performance of communication systems. Such a *noise* method is to be achieved by cryogenically-cooling wireless front-ends, and as a result, diminishing the impact of thermal noise. Despite the applications of such cooling in some existing systems (for details, see Section 2.2), we are not aware of any previous work that analyzed the underlying potential of such an approach, especially from a theoretical perspective. The novel contribution of the paper is the analysis of the impact of cryogenic cooling on:

- channel capacity in a system with and without interference and
- improving the performance of the communication system (in terms of lowering requirements on $E_{b}/N_{0}$) for a system with channels modeled by additive white Gaussian noise (AWGN) and Rayleigh channel models.

Apart from that, we provide an analysis of the temperature impact (in a continuous sense) on the symbol and bit error probabilities.

The remainder of the paper is structured as follows. We provide a brief introduction to cryogenics, together with the discussion of noise types in RF front-ends and examples of similar systems in Section 2. The impact of cryogenic cooling on the performance of a communication system is presented in Section 3. We analyze and discuss the behavior of such cooling in both AWGN and Rayleigh channel models and take into account interference from other systems. Section 4 discusses the limitations placed on systems employing the proposed cooling as a performance improving method, while possible deployments are discussed in Section 5. Section 6 concludes the paper.

## 2. Cryogenics Background

Cryogenics has developed as a branch of physics due to the efforts towards liquefying noble gases. It is fair to claim that the liquefaction of helium is the beginning of the physics of low temperatures [1]. Currently, cryogenics can be considered as a science located at the intersection of physics and engineering. In its core, this discipline studies low temperatures, i.e., ones that are below 90 Kelvins, together with methods of achieving and preserving them. From a more engineering-oriented perspective, it also investigates the behaviors and properties of materials at these temperatures and phenomena that are related to them, e.g., superconductivity, which results in virtually no electrical resistance of the conductor. The other phenomenon that is important from the communication point of view is the impact of temperature on thermal noise and the relation of cryogenics to this fact. The average power of thermal noise decreases along with temperature, due to the decreased mobility of electrical current carriers. Such a phenomenon may be used to improve signal-to-noise ratio (SNR), which is critical to the probability of the correct signal detection and channel capacity of communication systems.

### 2.1. Noise in the RF Part of a Receiver

Correct detection of any kind of signal in telecommunications is limited by noise. One of the most important types of noise recognized in electrical devices is thermal noise, which arises in each element of any electronic circuit. Other types of noise, such as shot noise or flicker noise (with power spectral density proportional to 1/f) deteriorate receiver performance as well; however, we will concentrate on thermal noise, as we are able to limit it by appropriate means.

It is well known that the power of thermal noise can be well characterized by the expression:

(2) $$N=k_{B}TB,$$

where $k_{B}$ is Boltzmann’s constant, *T* is temperature in Kelvins, and *B* is the bandwidth of the circuit. Let us recall the well-known fact that thermal noise power is proportional to the temperature at which the given circuit operates. Its influence has been thoroughly evaluated in radio astronomy. In [2], some quantitative values were given. The noise contribution of an L-band radio astronomy antenna was estimated to be about 3–4 K, whereas the noise temperature of amplifiers operating at cryogenic temperature levels was 2–4 K. The estimated total thermal noise of the system operating in the L-band was approximately 19 K.

It is important, however, to identify upfront some arbitrary limits related to thermal noise. One inevitable kind of noise that is present virtually everywhere is the thermal noise of the Universe, also known as the Cosmic Microwave Background (CMB). Despite its omnipresence, CMB radiation is the same and uniform across the whole Universe and cannot be associated with any source. The presence of such noise is due to the rapid expansion of the Universe during which the plasma and radiation filling, which were the main components of the early Universe *soup*, cooled enough for protons and electrons to form some of the simplest atoms, mainly hydrogen. That early phase of the Universe’s development is known as the epoch of recombination, and isotropic CMB radiation is its relic. The temperature of the Cosmic Microwave Background is approximately 2.726K. The existence of the CMB noise has an inevitable consequence. Mainly, since it is present everywhere, it cannot be neglected. Thus, even if we could, in theory, operate at absolute zero temperature, i.e., 0 Kelvins, we would still experience the impact of CMB. As a result, CMB should be perceived as a more real limitation than the absolute zero temperature. Although, as it is shown further in the paper, even at temperatures higher than 3K, the gain from cooling is still enormous.

### 2.2. Application of Cryogenic Front-Ends in Existing Systems

The application of high-temperature superconductive filters (HTSFs) and cryogenically-cooled low-noise amplifiers is well known in radio astronomy [2,3,4] to diminish thermal noise’s influence on the signal detection of very weak signals from deep space. A similar technology started to be cost-feasible in application to the base stations (BSs) of 3G systems already around the year 2008. A practical overview of cryogenic receiver front-ends in commercial wireless applications can be found in [5]. This was done on the basis of the developments made by Superconductor Technologies Inc. In [6], a 2-GHz band cryogenic receiver front-end composed of HTSFs and a cryogenically-cooled low-noise amplifier was presented, and its measured characteristics were shown. In [7,8,9], improvements in the coverage and capacity of CDMA wireless BSs employing cryogenic technology were discussed. Receiver front-ends using this technology are also offered for LTE (see [10] as an example).

## 3. Shifting the Limits due to Cryogenic Front-Ends

### 3.1. Additive White Gaussian Noise Channel

First, let us consider a communication system over the AWGN channel, which is ideal for modeling the impact of thermal noise. Considering the basic Shannon formula for additive white Gaussian noise channel capacity, Equation (1), and substituting the noise power with Equation (2) in it, we obtain a formula showing the channel capacity as a function of the receiver temperature, i.e.,

(3) $$C\left(T\right)=B\log\left(1+\frac{P_{rx}}{k_{B}TB}\right).$$

**Figure 1:**
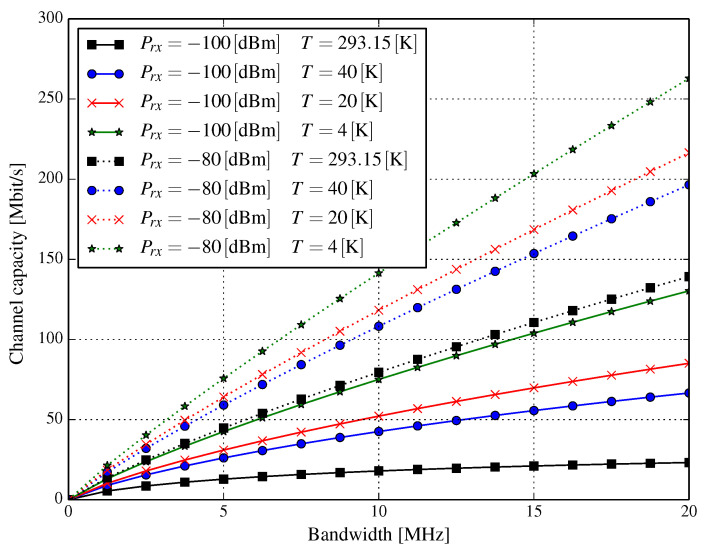
Shannon capacity for the additive white Gaussian noise channel with different noise temperatures versus system bandwidth.

Figure 1 shows the plots of channel capacity in Mbps versus channel bandwidth *B* for several temperatures of the RF receiver block, assuming the received power $P_{rx}=-100$ dBm and $P_{rx}=-80$ dBm. In the case when the received power is −100 dBm, we conclude that the Shannon capacity is around 23 Mbps for a 20 MHz system bandwidth at the temperature of 293.15 K, while it increases to approximately 85 Mbps when the temperature drops to 20 K. Such a temperature change results in more than a three-and-a-half-fold capacity increase. It can also be observed that if the temperature of the cryogenic front-end were at an extremely low level from the point of view of wireless communications, i.e., 4 K, the resulting capacity increase would be even greater (approximately seven-times larger than the capacity of the system working in the reference conditions). We have to admit that when the received signal is stronger, e.g., $P_{rx}=-80$ dBm, then the relative capacity gain is lower; however, it is still about two-fold for temperatures of 4 K and 20 K. Although the relative channel capacity (expressed in percentage with respect to the reference capacity at 293.15 K) decreases as the received power increases, we can see that the net capacity gain (in Mbps) rises slightly even when the received power increases. Bear in mind that when the temperature changes from 293.15 K to 20 K and the channel bandwidth is 20 MHz, the net capacity gain is approximately 62 Mbps and 77 Mbps for received powers −100 dBm and −80 dBm, respectively.

It is also important to note that in the case of a system with a receiver cryogenically cooled to the temperature of 4 K and the received power of −100 dBm, the capacity is almost the same as in a system in which the received power is at approximately −80 dBm, but without any additional cooling. Such an increase in capacity would barely be achievable without decreasing the temperature. Let us stress that although we show asymptotic limits, the performance of modern channel coding techniques, e.g., turbo codes [11] or low-density parity check (LDPC) codes [12], allows the performance of the overall system to be close to such limits (typically, the difference is about several hundredths of a decibel). Therefore, a comparison of various cooled-down systems at such limits is justified, provided that the resultant system would employ adequate coding techniques.

**Figure 2:**
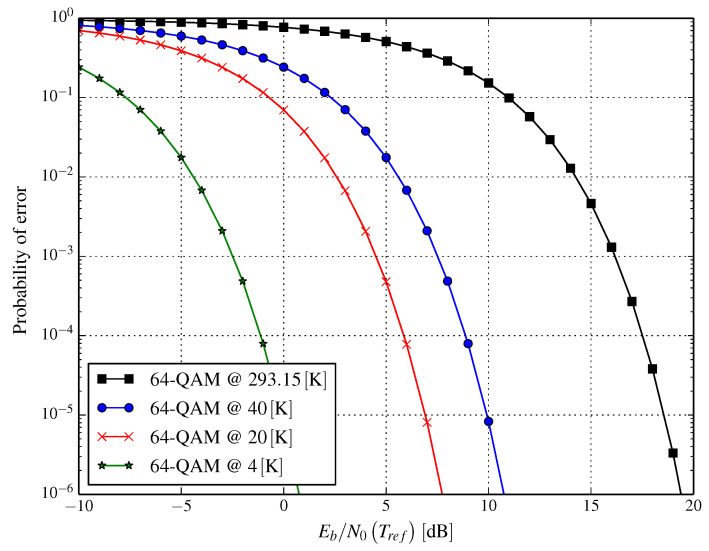
Symbol error probability for the 64-QAM and additive white Gaussian noise channel versus $E_{b}/N_{0}\left(T_{ref}\right)$ for several cryogenic cooling temperatures of the RF receiver part.

Cryogenic cooling of the RF part of the receiver has measurable consequences for the symbol error probability of the received data symbols. Considering quadrature amplitude modulation (QAM) in the presence of additive white Gaussian noise results in the expression for symbol error probability:

(4) $$\begin{array}{cc} \; & P_{S}^{M-QAM}=1-{\left(1-\frac{\sqrt{M}-1}{\sqrt{M}}\mathrm{erfc}\left(\sqrt{\frac{3q}{M-1}\frac{E_{b}}{N_{0}\left(T_{ref}\right)}\frac{T_{ref}}{T}}\right)\right)}^{2}, \end{array}$$

where $\mathrm{erfc}\left(\cdot \right)$ is the complementary error function, $M=2^{2q}$ is the modulation size, $E_{b}$ is the received energy per bit, and $N_{0}\left(T_{ref}\right)=k_{B}T_{ref}$ is the additive white Gaussian noise power density for the reference temperature $T_{ref}$. We conclude from Equation (4) that decreasing the noise power density due to lowering the temperature results in a gain in the required $E_{b}/N_{0}$ to be coarsely proportional to $10\log_{10}\left(T_{ref}/T\right)$ dB. This is illustrated in Figure 2. Assuming the reference temperature is 20 °C ($T_{ref}=293.15\;K$), the resulting gain is approximately equal to 18.3dB and 11.3dB for the operating temperatures of 4K and 20K, respectively.

From Figure 2, we can see that cooling the RF receiver part down to 20K from the room temperature of 293.15K results in an impressive gain of about 11.7dB in SNR per bit in the case of 64-QAM. The same gain in SNR per bit can be achieved for 16-QAM, as it depends on the change in temperature only.

### 3.2. Rayleigh Channel

Not taking into account phenomena specific to wireless communications, e.g., multipath propagation, is a disadvantage of the AWGN channel. A model reflecting such phenomena more accordingly is the Rayleigh flat fading channel, making it suitable for, e.g., modeling a single subcarrier channel in multi-carrier communications. According to [13], the bit-error probability for this channel can be expressed as:

$$P_{b}^{R}(T)=\{\begin{array}{lll} \left(1-2^{-q/2})F(q,T\right) & q=2k, & \;(5a)\\ F(q,T) & q=2k+1, & \;(5b) \end{array}$$

where *q* is the number of bits per symbol, k∈Z, and:

(6) $$F\left(q,T\right)=\frac{2}{q}\left(1-\sqrt{\frac{3q\gamma_{b}\left(T\right)}{2\left(2^{q}-1\right)+3q\gamma_{b}\left(T\right)}}\right),$$

where:

(7) $$\gamma_{b}\left(T\right)=\frac{E_{b}}{N_{0}\left(T\right)}.$$

**Figure 3:**
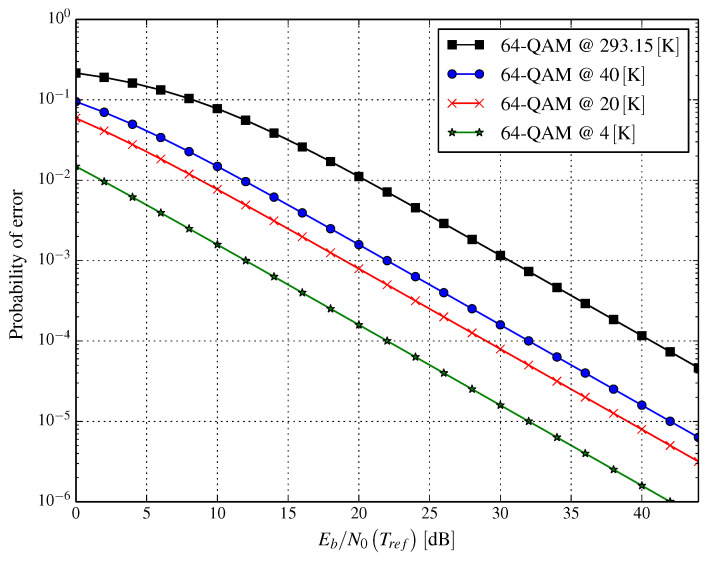
Bit error probability for 64QAM versus $E_{b}/N_{0}\left(T_{ref}\right)$ for several cryogenic cooling temperatures for the Rayleigh flat fading channel model.

Having this in mind, we can obtain similar results for the bit error probability for this channel model. They are displayed in Figure 3 in a very similar way as for the AWGN channel. Again, as expected, the gain obtained by decreasing the noise level due to cryogenic cooling to the temperature of 20K equals 11.7dB for the probability of the bit error being equal to $10^{-4}$. Very similar results can be obtained for 16-QAM. Since changing the modulation order does not impact cryogenic cooling, the gain in SNR per bit for a Rayleigh flat fading channel and 16-point QAM is also 11.7dB at a $10^{-4}$-bit error probability.

It can be concluded that both considered channel models, i.e., the AWGN channel and Rayleigh flat fading channel, are subject to a similar gain, i.e., the required minimum SNR per bit decreases by about 11.7dB. We would like to note that the gain of 11.7dB is (coarsely) proportional to the logarithm of the ratio of temperatures in reference and investigated conditions, i.e., $10\log_{10}\left(293.15/20\right)$. The results obtained for the AWGN channel are particularly promising for links in which thermal noise is dominant. It occurs for backhaul (BH) links, which often constitute a bottleneck in wireless systems when such a link is in use, e.g., utilizing a line-of-sight (LOS) transmission technique.

### 3.3. Temperature Impact

Figure 2 and Figure 3 visualize the error probabilities of the system. Such a presentation allows a simple comparison of the performance of systems operating in a similar wireless environment, but with or without a cryogenically-cooled receiver. At first glance, the presented plots seem to be shifted to the left. In reality, what happens is a reduction of the power spectral density of the noise ($N_{0}$) due to the cooling of the RF front-end, which should be interpreted as moving along the presented curve. The way in which we presented the results, however, allows us to compare the cryogenically-cooled system with one working in reference conditions.

**Figure 4:**
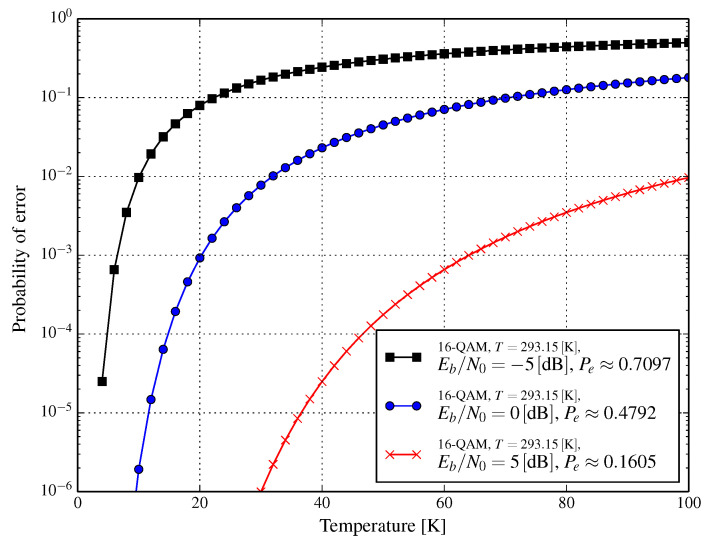
Symbol error probability for 16-QAM as a function of temperature of the RF receiver front-end for various values of $E_{b}/N_{0}$ achieved at $T_{ref}=293.15\;K$ and the additive white Gaussian noise channel.

**Figure 5:**
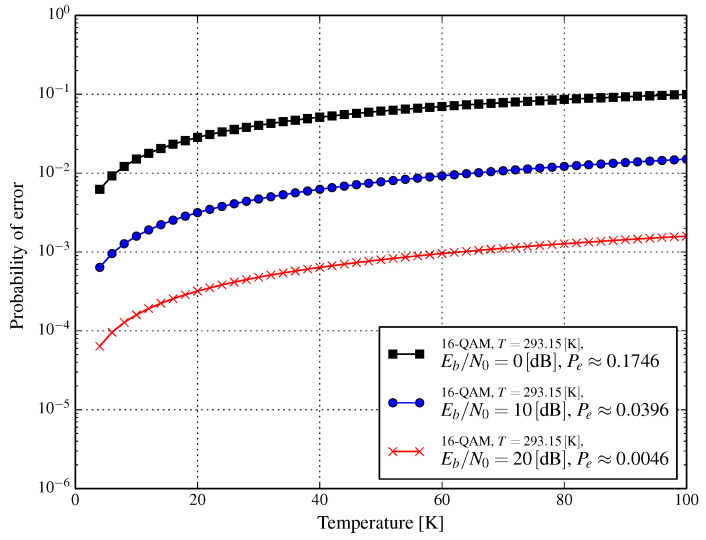
Bit error probability for 16-QAM as a function of temperature of the RF receiver front-end for various values of $E_{b}/N_{0}$ achieved at $T_{ref}=293.15\;K$ and the Rayleigh channel.

Unfortunately, in both Figure 2 and Figure 3, we cannot observe the continuous impact of temperature on error probability. This is addressed in Figure 4 and Figure 5 for AWGN and Rayleigh channels, respectively. In Figure 4, three curves are shown for the reference $E_{b}/N_{0}$ values that are obtained at $T_{ref}=293.15\;K$; these are −5dB, 0dB, and 5dB, the symbol error probability for these $E_{b}/N_{0}$ in the reference conditions being (approximately) 0.7097, 0.4792, and 0.1605, respectively. The impact of the temperature (in the range 4–100 K) of an RF front-end on error probability (presented in Figure 4) shows how much lowering the temperature changes the error probability. A similar effect is depicted in Figure 5, but for the Rayleigh channel instead.

### 3.4. Interference

In modern wireless networks, typical systems are rarely noise-limited. In fact, the chief limiting factor is interference from other cells and other users. In particular, the exact analysis of interference from various sources can be complicated. However, we can apply the simplifying assumption that interference originating from several, albeit independent, sources can be modeled as Gaussian noise with more or less flat power spectral density in the band of the transmitted signal. Thus, we can assume that the nature of interference is similar to the thermal noise. Denoting the interference power with respect to the signal power as α, i.e., I=αS, allows us to express the Shannon channel capacity as:

(8) $$C\left(T\right)=B\log\left(1+\frac{S}{k_{B}TB+\alpha S}\right).$$

The denominator in Equation (8) can be interpreted as temperature dependent signal-to-interference-plus-noise ratio (SINR). The rationale for such modeling of interference is that it covers the worst possible scenario, i.e., when the interference cannot be reduced by some more complex receivers, e.g., one that utilizes successive interference cancellation.

**Figure 6:**
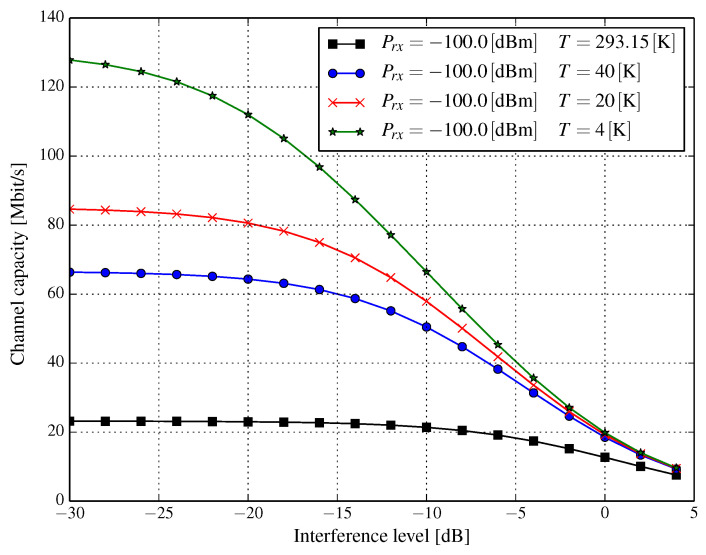
Channel capacity for a 20MHz bandwidth versus interference level α (dB) for several temperatures of the RF front-end and the received power $P_{rx}=-100.0\;\mathrm{dBm}$.

**Figure 7:**
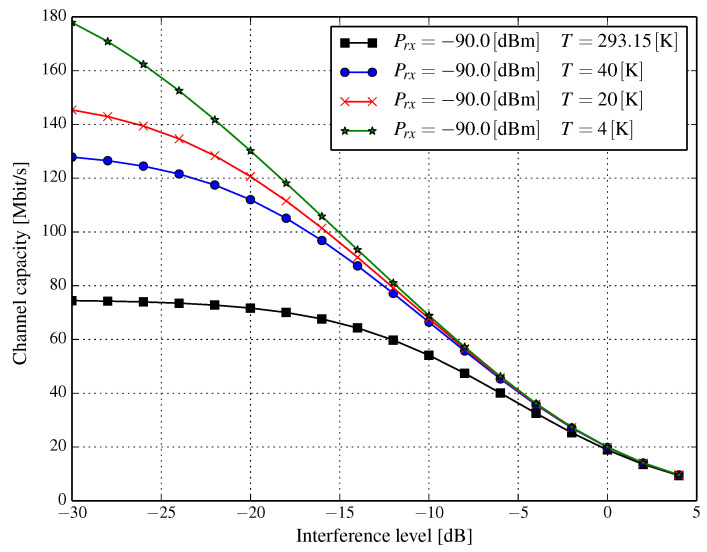
Channel capacity for a 20MHz bandwidth versus interference level α (dB) for several temperatures of the RF front-end and the received power $P_{rx}=-90.0\;\mathrm{dBm}$.

**Figure 8:**
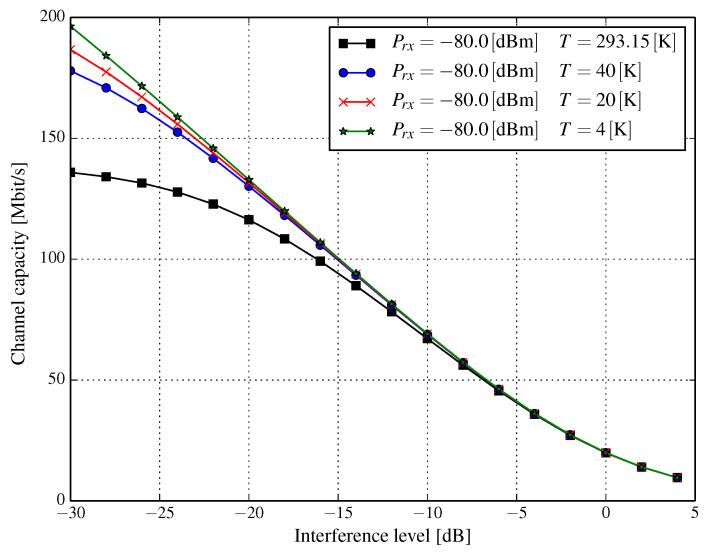
Channel capacity for a 20MHz bandwidth versus interference level α (dB) for several temperatures of the RF front-end and the received power $P_{rx}=-80.0\;\mathrm{dBm}$.

Figure 6, Figure 7 and Figure 8 show the resulting capacities versus interference level α=I/S, expressed in dB for a channel of a 20MHz bandwidth. We observe that the interference level for several received signal power levels has a substantial influence on channel capacity. When the received signal power is very low, e.g., $P_{rx}=-100\;\mathrm{dBm}$ (q.v. Figure 6), a capacity gain of approximately 50 percent can be achieved even for signal-to-interference ratio (SIR) at the level of 0dB, whereas it is almost three-fold for SIR=10dB (α=−10dB) when the temperature drops from 293.15K to 20K. Regardless of the received power, as long as the interference level falls, the channel capacity increases. However, at some interference level, the increase of the channel capacity stabilizes, e.g., for $P_{rx}=-100\;\mathrm{dBm}$ and T=20K (Figure 6), this happens for approximately α=−20 dB for which the channel capacity does not increase significantly above 80–85 Mbps. This phenomenon is due to the fact that when the interference level becomes very low, its value can be comparable with that of the thermal noise; albeit, depending on the temperature of the cryogenically-cooled down wireless front-end and the received power, such a capacity stabilization point can be found at different interference levels. We can see this by comparing Figure 7 ($P_{rx}=-90\;\mathrm{dBm}$ and T=20K) with Figure 6 ($P_{rx}=-100\;\mathrm{dBm}$ and T=20K). In the latter (Figure 6), the stabilization of channel capacity occurs, as said, somewhere around SIR=20 dB, while in the former (Figure 7), the capacity seems to be increasing even when SIR is around 30 dB (α=−30dB). As has already been stated, in conditions when interference significantly dominates the thermal noise, i.e., αS≫N, a significant lowering of the temperature has a visible impact when SIR is really high, e.g., at the level of 25 dB (see Figure 8). This limits the applicability of the proposed solution in situations when a considerable amount of *distortion* comes from interference.

## 4. Limitations of Cryogenics

So far, we have analyzed the influence of cooling temperature on channel capacity in various situations. However, there is a certain limitation involved in cryogenic cooling that is due to the antenna noise. Unfortunately, typical communication systems used on Earth are equipped with antennas directed at the horizon (in the case of cellular systems, this is not even the case, since in such deployments, antennas tend to be directed even *below* the horizon line in order to control the cell size). The problem that arises from the orientation is that at antenna terminals, there is available noise of the equivalent temperature equal to the temperature of the source at which the antenna is *looking*. If the antenna is directed at the elevation angle $0^{\circ }$, i.e., horizontally, the approximate noise equivalent temperature at its terminals would be (3+293)/2=148 K. Half of this noise coming from the Earth has an approximate temperature of 293 K, and the other half coming from the sky has a temperature of approximately 3 K. This noise, unfortunately, adds up to the noise of the system, degenerating the performance of the latter. Assuming that the temperature of the system can be lowered to 3 K, the resultant noise temperature would be about 148+3=151K, which gives the approximate gain of $10\log\left(\frac{293}{151}\right)\approx 3\;\mathrm{dB}$ when compared to the system without cooling. Details rationalizing this statement are included in Appendix A.

We would also like to highlight here that the aforementioned 3dB gain limitation is only encountered on Earth. Thus, cryogenic cooling could be significantly more beneficial in space communication, e.g., in the case of Earth-Mars communication, etc.

**Figure 9:**
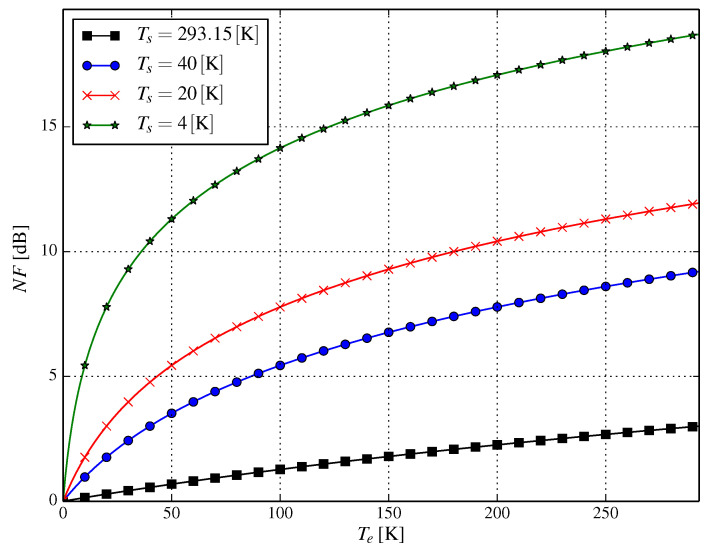
The noise figure as a function of temperature.

Another important factor that may limit the system employing cryogenic cooling is the NF of the amplifiers’ chain. In an idealized world, the input signal at the receiver side is amplified in order to allow detection without the corruption of the input signal. In the real world, sadly, the input signal is additionally corrupted by the amplifier itself. Such signal quality degradation can be described by the noise figure; in fact, the signal-to-noise ratio is degraded by the value of the noise figure parameter. The impact of the noise figure can be viewed as introducing additional noise into the system. The noise figure as a function of temperature can be expressed as:

(9) $$NF\left(T_{s},T_{e}\right)=10\log\left(\frac{T_{e}+T_{s}}{T_{s}}\right),$$

where $T_{s}$ is the temperature of the source, i.e., the temperature (in Kelvins) of the object the antenna is directed at, and $T_{e}$ is the equivalent noise temperature of the source impedance, i.e., the temperature of the receiver. For example, for $T_{s}=150\;K$ and $T_{e}=20\;K$, the theoretical value of the noise figure is approximately 0.54dB. In Figure 9, we show plots of NF (described by Equation (9)) for various values of $T_{e}$ and $T_{s}$. In fact, cryogenic cooling has (and must have) a limited impact on terrestrial communications.

Despite all of the above, it should be mentioned that low-noise amplifiers that can operate at cryogenic temperatures are already commercially available. For example, in the 1.5GHz–6GHz band, a low noise amplifier with a gain of 26dB and the NF of 0.031dB is available (see [14] for details), while the typical values of noise figure (NF) are 5–7 dB.

Therefore, it is not a limitation that could not be overcome.

## 5. Cryogenic Possibilities

Cryogenic cooling of receiver front-ends can be potentially beneficial in terms of capacity and the probability of correct detection. In this section, we focus our attention on the possible deployments of network entities equipped with such receivers. We would also like to highlight that despite the incredibly fast growth and development of the electronic industry, in our opinion, the implementation of such cooling in mobile stations is neither technologically nor economically feasible in the near future. Cooling requires additional power, which is a critical factor in battery-powered devices. Therefore, we only discuss cryogenic cooling in entities that convey rather vast amounts of traffic.

According to the results shown in Figure 2 and Figure 3, the SNR per bit gain could be substantial. This can have two mutually-exclusive outcomes:

1. Reducing the transmit power and, as a result, reducing $P_{rx}$, while preserving the capacity of the system or
2. Increasing channel capacity.

Of course, we can also control the temperature of the RF front-end, so that the result would be a mix of capacity gain and TX power reduction. The question about whether it should be more capacity-oriented or energy reduction-oriented can be decided dynamically according to the current requirements of the network. Such control could be utilized to adapt the whole network to long-term fluctuations in throughput demand, load, etc.

Another aspect that is inextricably connected with using less transmit power is battery life. Since the transmit power (of a mobile terminal) could be reduced when the receiving node (a base station) has its front-end cooled down, then the time between recharging batteries could be extended and, as a result, the battery life of mobile terminals, as well. Certainly, such battery life extension could be marginal, but nevertheless possible, even in devices that use most of their battery-stored energy for other purposes, e.g., powering screens of smart phones, let alone devices that consume virtually all of their power solely for communication. In particular, such an application of cryogenic cooling could be more important to the operation of machine-type communications (MTCs) utilized by the Internet of Things (IoT). This could be especially useful in networks that need to gather information from thousands of sensors, actuators, and other embedded, but Internet-aware electronics. In such scenarios even the Earth-limited gain of cryogenic cooling achieved at 150 K, i.e., the gain of 3 dB, may increase the number of simultaneously-connected devices by a factor of two (assuming a two-fold capacity increase), which is the equivalent of deploying a second receiving station. The apparent advantage of cryogenic cooling is that it may be used depending on the network load, while deploying another station is rather permanent. The 3 dB gain provides a way to improve the capacity of existing systems in situations when neither frequency nor concrete (e.g., new base stations) resources can be added to the system.

The very last and slightly more sophisticated benefit that could be gained from cryogenic cooling is related to the spectrum leasing of relay nodes (RNs) in networks where RNs operate in the in-band mode. Considering a typical relay-enhanced network that is composed of macro base stations at the centers of its cells and capacity-increasing relays located at cell edges and assuming that in each cell, the BS is equipped with RF front-ends whose operating temperature can be lowered to cryogenic levels, we can speculate on some consequences of such a deployment. Furthermore, in more rapidly-changing environments, resources assigned to RNs are chosen dynamically rather than statically. Since this is typically done by a scheduler, then the scheduler could easily take into account another system dimension, i.e., the temperature of wireless front-ends, and optimize resource scheduling to an even greater extent. This could possibly reduce intra-cell interference, especially the one from the BH link to the access link (as we assume that interference from the access link to the backhaul link is negligible), and help increase the frequency reuse factor. On the other hand, the available capacity at the access uplink could be increased without assigning more time or frequency resources to it, since the interference from backhaul links would be limited as a result of the reduced transmit power of relay nodes and user equipments (UEs).

## 6. Conclusions

The application of cryogenic cooling to the RF part of receivers in crucial nodes of communication systems could be one of the possible means of meeting the increasing traffic demands that are to be expected in the future, and it can have several positive consequences for deployments of such systems. First, it substantially increases theoretical channel capacity for AWGN and Rayleigh channels when thermal noise is a dominant type of distortion. The gains remain further significant for channels with interference. The main nodes where cryogenic cooling can be applied are backhaul links and base station receivers. Secondly, cryogenic cooling enables signal detection at much lower power levels. On the one hand, it means the cell size could be enlarged (see also [7,8,9]), which is particularly advantageous for communication in the higher frequency ranges and macro cells in sparsely-populated areas. On the other hand, the ability to detect signals received by a base station at lower power levels enables the decrease of the power emitted by mobile terminals, increasing their power efficiency and time intervals between necessary battery loading. This possibility is also in agreement not only with the general rules of green communication, but also with machine-type communications, where the operational time of the devices could be extremely long. Finally, despite showing only asymptotic limits, there are no visible contraindications to using cryogenic cooling, channel coding, and other techniques together in order to achieve these limits.

One important aspect that we did not discuss in this paper is the economic feasibility of cryogenic cooling in wireless network entities. We are fully aware of this problem; however, we view it as being outside the scope of the paper. 

## Appendix A. Antenna Temperature

This Appendix explains why the use of cryogenically-cooled receivers has a limited impact on terrestrial communications.

According to Nyquist [15], and not considering quantum effects, the power of the thermal noise can be approximated by:

(A1) $$P=k_{B}TB,$$

where $k_{B}$ is the Boltzmann constant, *T* is the temperature of the system in Kelvins, and *B* is the bandwidth in Hz. (In fact, taking into account quantum effects that occur at both low temperatures and high frequencies requires scaling Equation (A1) by $n\left(f\right)=\frac{hf/k_{B}T}{\exp(hf/k_{B}T)-1}$, where *h* is Planck’s constant.) Now, according to [16], the temperature of the system is given by:

(A2) $$T_{sys}=T_{A}+T_{AP}\left(\frac{1}{\epsilon_{1}}-1\right)+T_{LP}\left(\frac{1}{\epsilon_{2}}-1\right)+\frac{T_{R}}{\epsilon_{2}},$$

where:

- $T_{A}$ is the antenna noise temperature (in Kelvins),
- $T_{AP}$ is the antenna physical temperature (in Kelvins),
- $\epsilon_{1}$ is the antenna (thermal) efficiency ($0\leq \epsilon_{1}\leq 1$),
- $T_{LP}$ is the line (waveguide) physical temperature (in Kelvins),
- $\epsilon_{2}$ is the line (waveguide) efficiency ($0\leq \epsilon_{1}\leq 1$), and
- $T_{R}$ is the receiver temperature (in Kelvins), which is, in turn, given as:
  (A3) $$T_{R}=T_{1}+\frac{T_{2}}{G_{1}}+\frac{T_{3}}{G_{1}G_{2}}+\ldots ,$$
  where $T_{1},T_{2},\ldots$ are the temperatures of the consecutive stages of a receiver and $G_{1},G_{2},\ldots$ are the gains of these stages.

Let us suppose that the system is operating at a cryogenic temperature, e.g., T=3K, i.e.,

(A4) $$T_{AP}=T_{LP}=T_{1}=T_{2}=\ldots =3\;K.$$

Assuming line and antenna efficiencies to be 0.99 and the receiver to be composed of two stages of low-noise amplifiers (LNAs) with a noise temperature of 4K and gains 20 dB

(A5) $$3\left(\frac{1}{0.99}-1\right)+3\left(\frac{1}{0.99}-1\right)+\left(3+\frac{3}{100}\right)\approx 3.09\;K.$$

(Values used in Equation (A5) are made so extremely good on purpose.) This results in the overall system temperature:

(A6) $$T_{sys}=T_{A}+3.09.$$

The antenna temperature, on the other hand, is given by:

(A7) $$T_{A}=\frac{1}{\Omega_{A}}\int_{0}^{\pi }\int_{0}^{2\pi }T_{s}\left(\theta ,\phi \right)P_{n}\left(\theta ,\phi \right)d\Omega ,$$

where:

- dΩ is a solid angle differential in spherical coordinates, i.e., sinθdθdϕ,
- $\Omega_{A}$ is the antenna beam solid angle ($0<\Omega_{A}\leq 4\pi^{2}$),
- $P_{n}\left(\theta ,\phi \right)$ is a normalized antenna beam pattern, and
- $T_{s}\left(\theta ,\phi \right)$ is the brightness temperature of sources as a function of the solid angle.

In typical deployments, antennas work in an environment where half of the noise comes from the ground that has a temperature of around 300K. Assuming that the remaining part of the hemisphere (defined by angle Ω) is responsible for only 3K of the noise temperature, then for an isotropic antenna ($P_{n}\left(\theta ,\phi \right)=1$, $\Omega_{A}=4\pi^{2}$), the antenna temperature is about 150K, resulting in the average system temperature being:

(A8) $$T_{sys}\approx 150\;K+3.09\;K=153.09\;K.$$

When compared to the reference temperature ($T_{ref}\approx 300\;K$), this gives a gain of:

(A9) $$10\log\left(\frac{T_{ref}}{T_{sys}}\right)\approx 10\log\left(2\right)\approx 3\;\mathrm{dB}.$$

The choice of an isotropic antenna was made to simplify the calculations and establish somewhat boundary results. In the case of more realistic antennas, the result might be slightly different depending on the specific antenna beam pattern.
